# Catheter-based Minimally Invasive Evacuation of Extensive Spinal Epidural Abscess: A Technical Report

**DOI:** 10.7759/cureus.4649

**Published:** 2019-05-13

**Authors:** Daniel J Denis, Pierre-Olivier Champagne, Haydn Hoffman, Tianyi Niu, Daniel C Lu

**Affiliations:** 1 Neurosurgery, Ochsner Medical Center, New Orleans, USA; 2 Neurosurgery, Enfant-Jesus Hospital, Laval University, Quebec, CAN; 3 Neurosurgery, University of California, Los Angeles, Los Angeles, USA

**Keywords:** radiopaque, epidural abscess, septic facet joint, minimally invasive

## Abstract

Surgical treatment of extensive spinal epidural abscess (SEA) usually involves multilevel exposure of the dural sac with subsequent risk for iatrogenic instability. A minimally invasive technique using an epidural catheter inserted through a limited approach for distant irrigation and drainage of the abscess represents an interesting alternative. Most described techniques involve blind placement of the catheters, with the potential risk of damage to the spinal cord and incomplete abscess drainage. We present and analyze a new technique used in two cases of SEA. Those were successfully treated using a minimally invasive approach supplemented with fluoroscopically-guided catheter drainage. We suggest that fluoroscopic placement of the catheter is a safe and effective method that offers a more focused and potentially safer way to proceed to this technique.

## Introduction

A spinal epidural abscess (SEA) is an expanding suppurative infection in the spinal canal between the dura mater and the vertebral periosteum. Most SEAs are associated with spondylodiskitis while a minority can be found next to a pyogenic facet joint (PFJ) or without concomitant spinal infection [1-7].

To reduce operative morbidity, multilevel SEAs can be drained through a limited open approach allowing blind epidural catheter irrigation and aspiration [8, 9]. Limited exposure of the dural canal during SEA surgery offers a greater chance for stability preservation and reduced morbidity. Such advantages are considered significant in this population of patients often affected by morbid obesity, diabetes, and end-stage renal disease [6, 10]. However, for extensive abscesses, when the epidural catheter is blindly advanced to attempt drainage of distant epidural fluid collection, incomplete SEA evacuation may ensue.

To ensure correct positioning in the epidural space, fluoroscopically-guided catheter placement has been suggested in the literature [8, 11], but to our knowledge no case has been reported. We report one case of lumbar and one case of cervical SEA extending over multiple spinal levels that were evacuated using a fluoroscopically-guided catheter through a limited exposure to the spine. Visualization of the catheter with fluoroscopy was a key factor in ensuring complete evacuation of the SEA.

## Technical report

Case #1

Presentation

**Figure 1:**
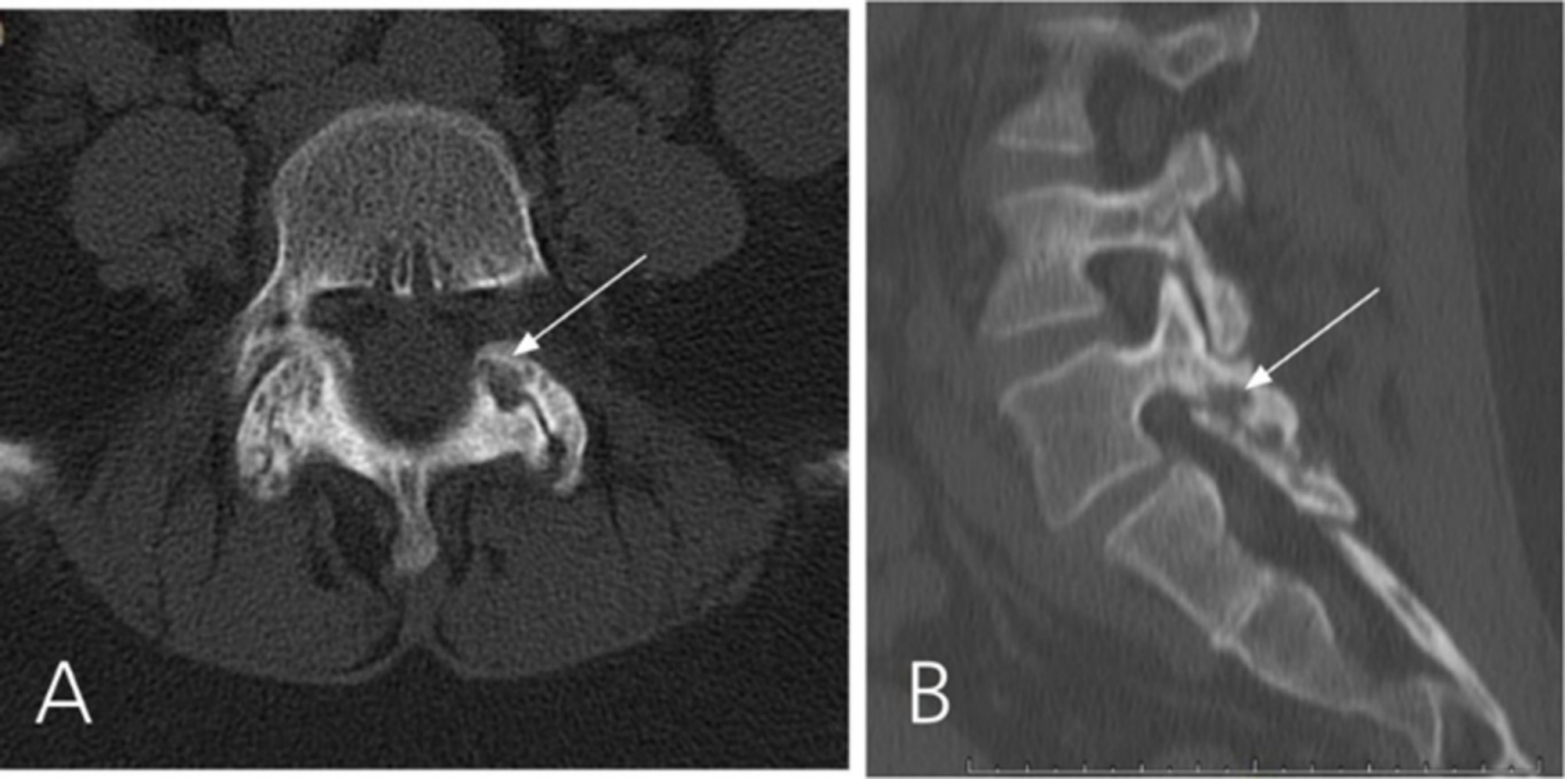
CT scan of Case #1 CT scan showing left L5-S1 facet erosive bony changes (white arrows) suggestive of a septic arthritis of the facet joint. A: axial image. B: sagittal image.

**Figure 2:**
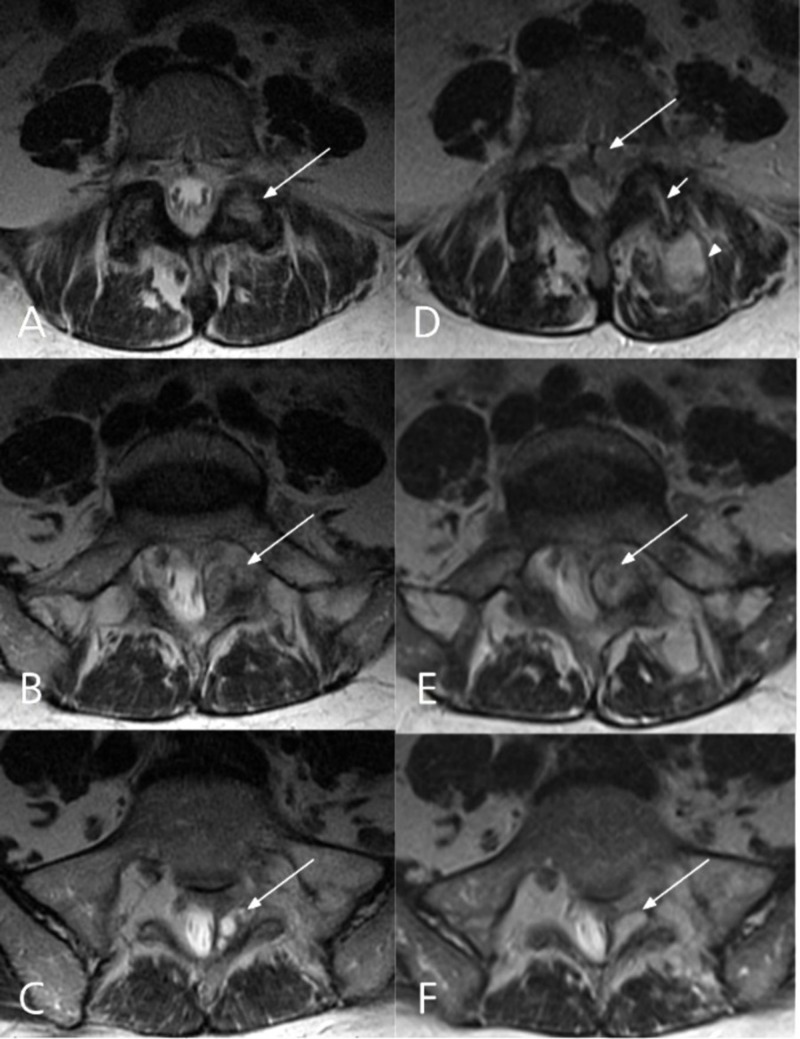
Comparative axial T2-weighted MRI images 2 months (A, C, E) and 15 months (B, D, F) after initial presentation The septic arthritis of the left L5-S1 facet joint has complicated into an epidural abscess (D). A: Right L5-S1 facet involvement with inflammatory change (arrow). B-C: Complex multi-cystic lesion originating from left L5/S1 facet (arrows). D: Isointense posterior and anterolateral epidural collections (long arrow), widening of the left L5-S1 facet joint (short arrow) and paraspinal muscle inflammatory changes (arrow head). E: The cystic lesion has increased in size (arrow). F: The content of the cystic lesion has changed and is now more homogenous (arrow).

A 53-year-old female without comorbidities, immunosuppression, or recent infection presented with back pain and left leg pain, without neurological deficit or fever. Based on imaging, (Figures 1, 2) she was diagnosed with a left L5-S1 complex synovial cyst and was treated conservatively with physical therapy and steroid epidural injections.

**Figure 3:**
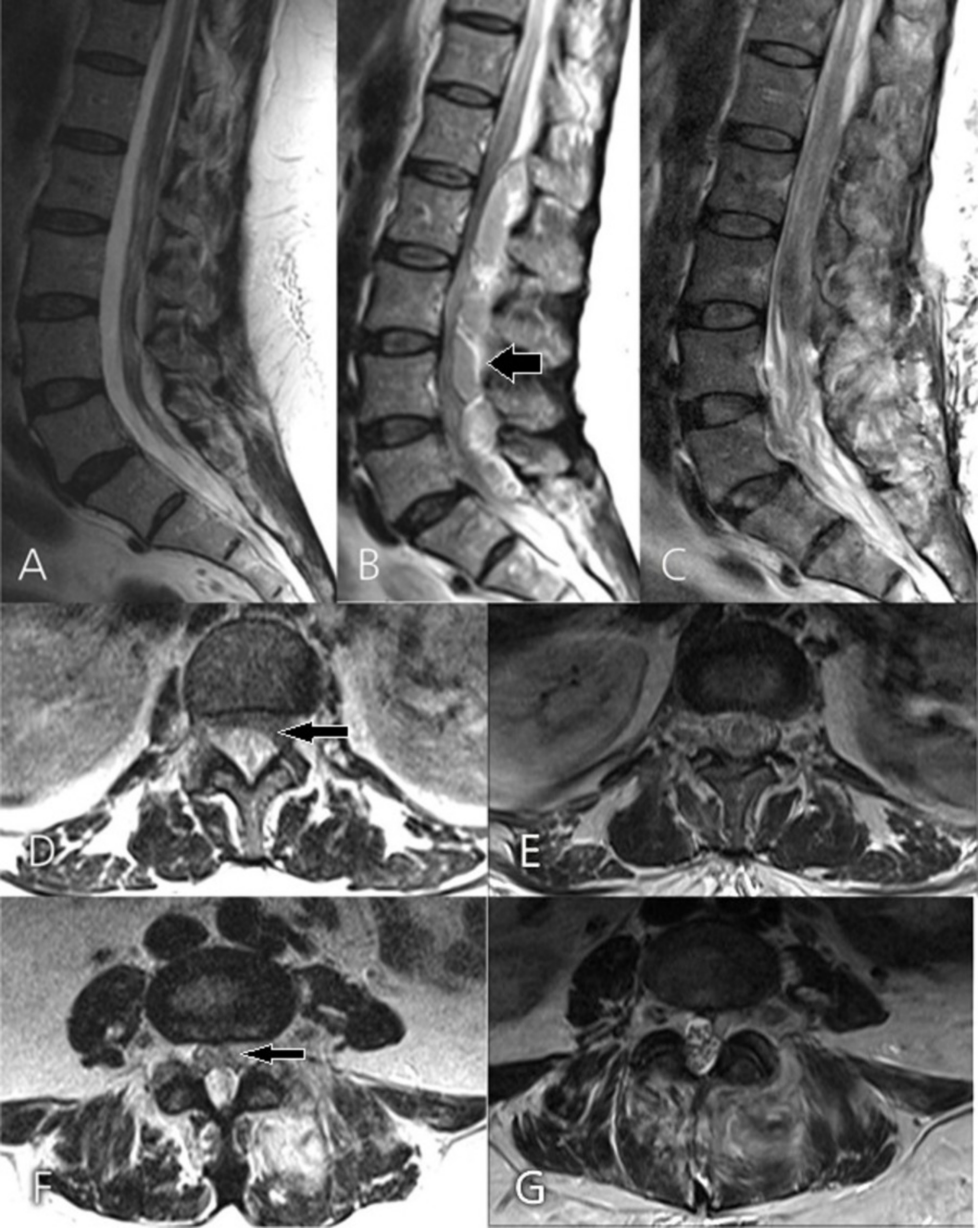
Comparative pre- and postoperative MRI images of Case #1 Comparative sagittal T2-weighted MRI at 2 months (A) and 15 months (B) after the initial presentation, and one day after surgery (C). B: Epidural abscess extending from L1 through S1 (arrow). C: Postoperative image demonstrating complete abscess evacuation. Axial T2-weighted MRI at L1-2 (D) and L4-5 (F) taken 15 months after the initial presentation (arrow representing the epidural abscess) and corresponding axial cuts after surgery (E and G).

Fifteen months later, she presented with increased radicular and low back pain. Her temperature was 38.3°C. She had left hip flexion weakness (4+/5) but no other neurological deficit. She had leucocytosis and an increased serum C-reactive protein level. A lumbar spine MRI showed a large, multilobulated, irregularly enhancing dorsal epidural fluid collection extending from L1 through S1 causing severe spinal canal stenosis highly suspicious of SEA. The cystic lesion arising from the left L5-S1 facet joint was increased in size when compared to the previous MRI from 15 months ago (Figures 2, 3). A CT-guided aspiration of the epidural collection was performed and intravenous vancomycin with piperacillin-tazobactam was started empirically. On the next morning, she developed acute saddle anesthesia and urinary incontinence. She was then taken to the operating room emergently for surgical decompression and drainage of the epidural abscess.

Operative Technique

**Figure 4:**
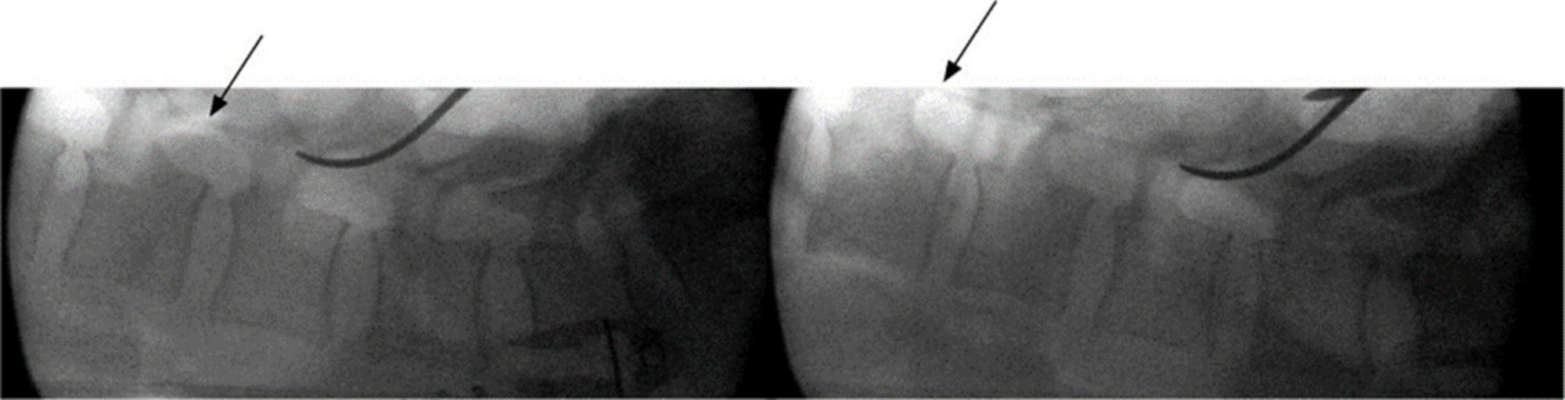
Intraoperative lateral fluoroscopy view of the lumbar spine showing advancement of the radiopaque 5F urologic catheter tip in the epidural space up to L1-2 (arrows)

A 3.5 cm midline incision was made targeting L4 to S1. Exposure of the left L5-S1 facet joint revealed yellowish creamy pus oozing from the facet joint and a specimen was obtained for cultures. A decompressive L5 laminectomy was carried out, and surgical debridement of the left L5-S1 facet joint did not reveal the presence of a synovial cyst. A 5 French open-ended barium impregnated ureteral catheter (Cook®, Bloomington, IN) was inserted rostrally into the posterior epidural space using a Penfield No. 3 dissector. The catheter tip was advanced under the lamina of L1 under fluoroscopic guidance (Figure 4), and the epidural space was irrigated. The length of the inserted ureteral catheter was marked and the measurement was transposed to a pediatric nasogastric (NG) tube for insertion in the epidural space. Saline and bacitracin were used for irrigation and additional pus was aspirated through the nasogastric tube. The dura was exposed and no residual fluid collection or pus was observed.

Postoperative Outcome

**Figure 5:**
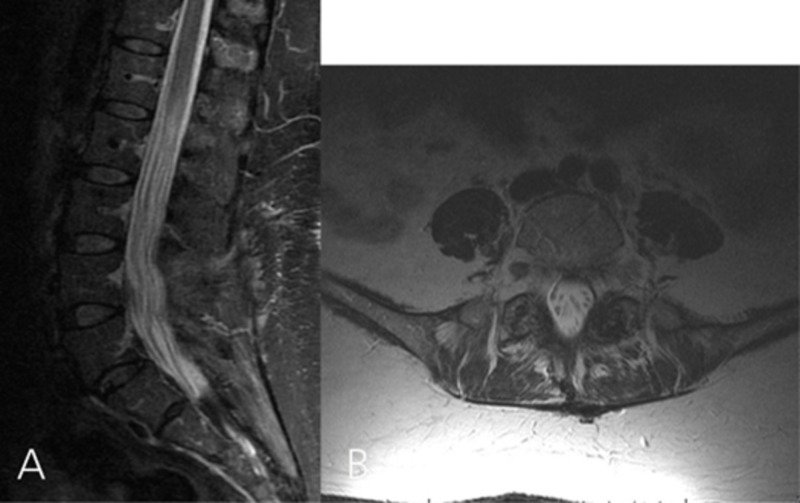
Repeated lumbar MRI done 11 months after the surgery. A: Sagittal T2-weighted image showing no residual canal stenosis. B: Axial T2-weighted image showing that the cystic lesion involving the left L5-S1 facet complex has resolved completely

A postoperative lumbar MRI showed resolution of the spinal canal stenosis (Figure 3C). Cultures grew methicillin-sensitive *Staphylococcus aureus*. The patient received a six-week course of intravenous oxacillin. Three weeks after surgery she was voiding spontaneously and her saddle anesthesia had completely resolved. Eleven months after surgery she experienced no further neurological deficits or pain in the lumbar area, and repeat lumbar MRI showed no residual stenosis (Figure 5).

Case #2

Presentation

**Figure 6:**
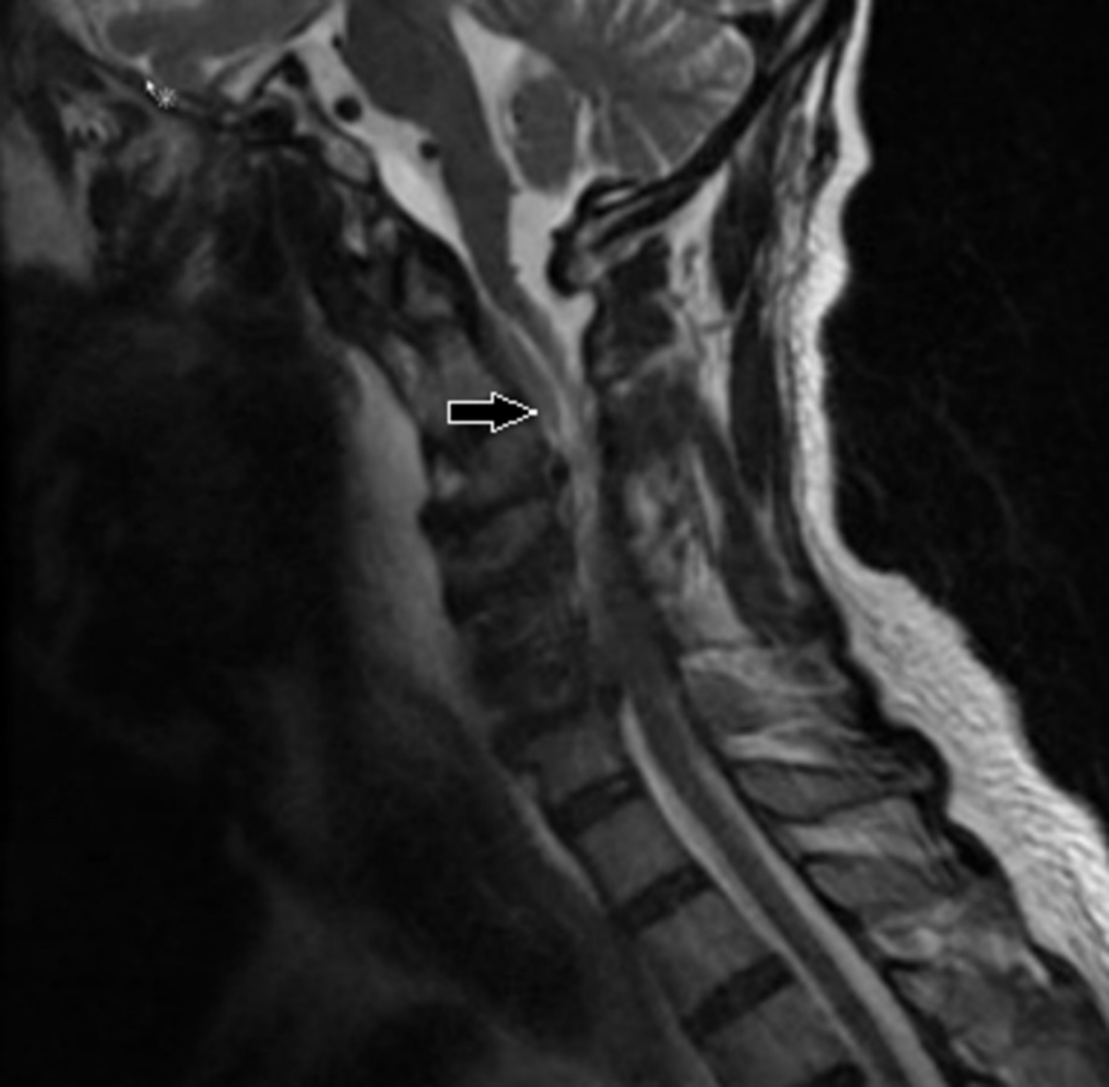
T2-weighted cervical spine MRI in the sagittal plane showing an anterior epidural collection spanning from C2 to C7 with associated mass effect on the spinal cord (arrow)

A 75-year-old man with a history of diabetes, hypertension, coronary artery disease, and prostate cancer presented to the emergency with confusion and right upper extremity pain radiating from the neck. His initial temperature was 37.4°C and a physical examination revealed proximal right upper extremity weakness (0-1/5). A head CT followed by MRI were unremarkable. A cervical spine CT showed mottling of the C7 vertebra, and subsequent MRI of the cervical spine demonstrated a C5-C6 spondylodiskitis with a compressive anterior epidural collection compatible with an abscess spanning from C2 to C7 (Figure 6). His blood cultures were positive for methicillin-resistant *Staphylococcus aureus*, which was treated with a course of vancomycin.

Operative Technique

**Figure 7:**
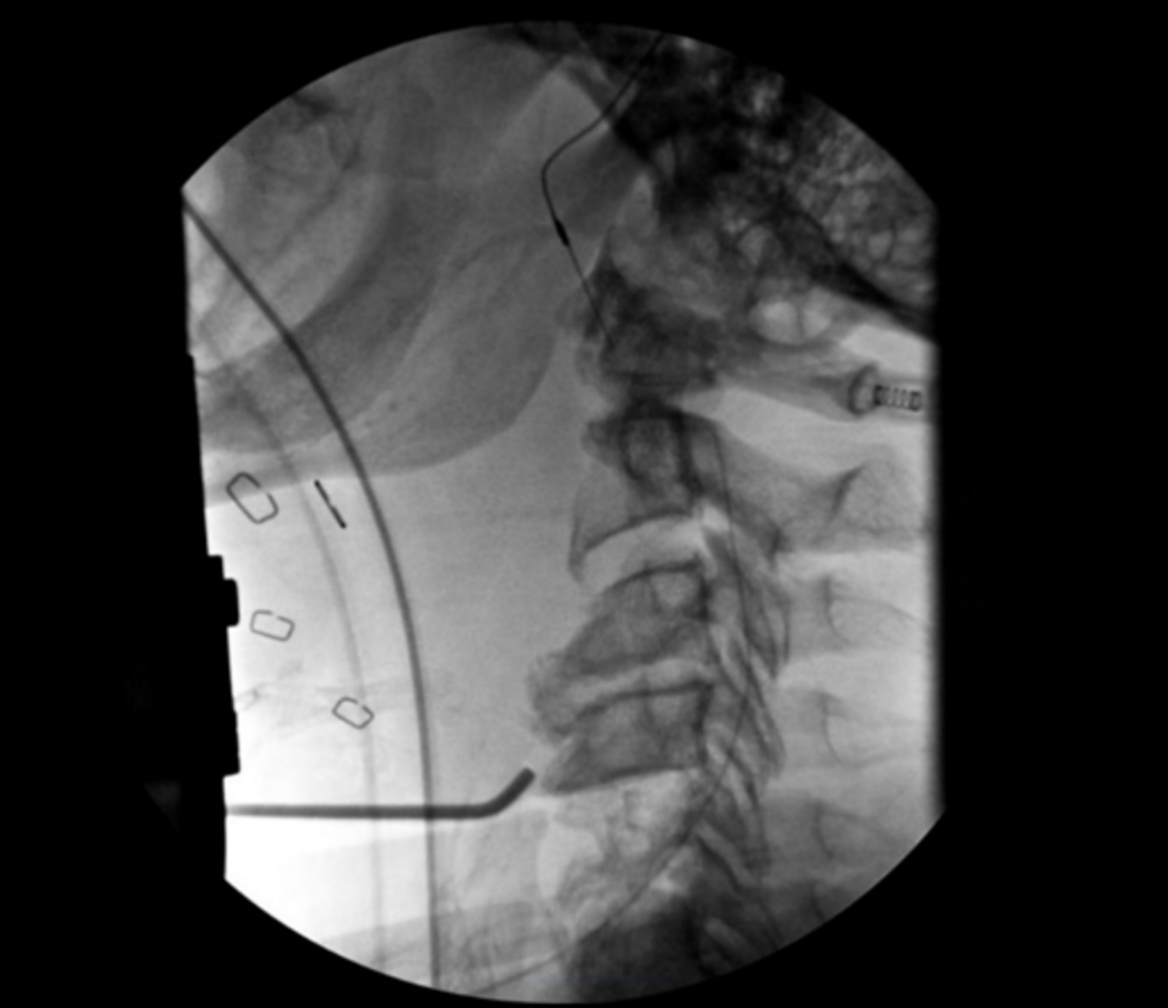
Peroperative fluoroscopic image showing good placement of the epidural catheter in regard to the upper portion of the C2 vertebra

The patient was brought to the operating room (OR) for an anterior decompression and instrumented fusion. Through a Smith-Robinson anterior approach, a C4-5 and C5-6 discectomy and C5 corpectomy were performed. The superior endplate of C6 was debrided from the infected tissue until healthy bleeding bone was reached. After aspiration of the local epidural abscess, a 7 French TLS drain (Stryker®, Kalamazoo, MI) was inserted under fluoroscopy up to the C2 level then downward to the C7 level (Figure 7). With the catheter in place, the remainder of the abscess was drained using gentle manual aspiration and irrigation with a 5 cc syringe. Instrumentation with cage and plate was then carried out in the usual fashion.

Postoperative Outcome

**Figure 8:**
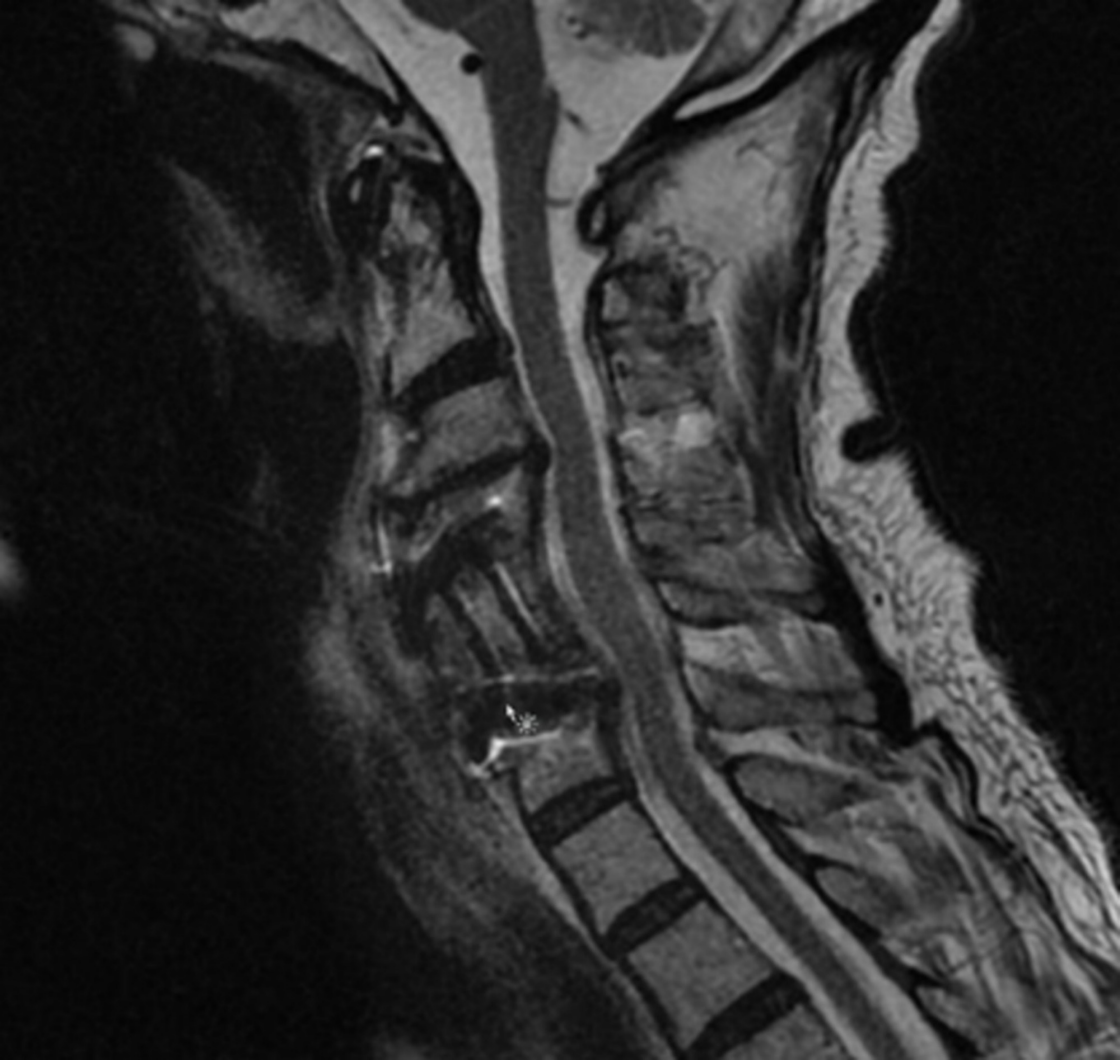
Postoperative T2-weighted cervical spine MRI in the sagittal plane showing expected postoperative changes with no residual compression of the dural sac

The patient recovered well postoperatively with improvement of mental status. However, his strength remained similar with weakness (0-1/5) of the right biceps and deltoid, consistent with right C5 palsy. Postoperative MRI showed a good resolution of the abscess (Figure 8). The patient was later discharged for rehabilitation. Of note, a percutaneous gastrostomy had to be put by a gastroenterologist due to postoperative dysphagia, which improved on follow-up.

## Discussion

The incidence of SEAs varies from 0.2-2 cases per 10,000 hospital admissions and is likely rising due to increased population age, intravenous (IV) drug use and complications from therapeutic spinal interventions [12].

In both cases presented, secondary epidural abscess was thought to have spread from a concomitant pyogenic infection. Case #2 presented with cervical spondylodiskitis with anterior SEA causing radiculopathy and myelopathy. The SEA in Case #1 was associated with a lumbar PFJ.

Pyogenic facet joint (PFJ) is found in 4% of all hematogenous pyogenic infections of the spine and is associated with concurrent extraspinal infection, diabetes mellitus, liver disease, end-stage renal disease, morbid obesity, intravenous drug abuse, alcohol abuse, and chronic steroid use [6, 10]. Other etiologies reported are infective endocarditis [2], acupuncture treatment [1], urinary tract infection [4], and facet injections [5, 7]. Patients commonly present with acute onset of back pain, radiculopathy, fever or paresis. The organisms more frequently found in tissue or blood cultures are *Staphylococcus* *aureus*, followed by *Streptococcus sp*. and gram-negative rods [6, 10].

The duration of symptoms before diagnosis of a pyogenic facet joint infection can vary from a few days [3] to several months [6, 13]. In our case, the indolent presentation was not the only factor contributing for the delayed diagnosis: the patient did not have risk factors, immunosuppression or recent infection, and was misdiagnosed with a synovial cyst based on MRI. Initial CT-scan displayed erosive bony changes of the left L5-S1 facet joint (Figure 1). However, the appearance of a synovial cyst on CT is not typically associated with bone erosion but it may present with sclerosis, gas or calcifications in the cyst capsule [14]. In contrast to synovial cysts, bone erosion is reported frequently with PFJ [6, 15]. On T2-weighted MRI scans, PFJ demonstrates widening of the facet joint space as well as erosion and swelling (Figure 2D). Associated inflammatory changes in the epidural space or adjacent paraspinal muscles can be distinguished with gadolinium-enhanced T1-weighted MRI. Gallium-67 scintigraphy can be used when MRI is contraindicated and has good sensibility and sensitivity [4].

Mild symptomatic PFJ without significant SEA can be treated with percutaneous CT drainage followed by intravenous antibiotics [16]. The success rate for percutaneous drainage and antibiotics is 85% while it is 71% for antibiotics alone [6]. Surgery is indicated for facet joint infections complicated with SEA causing significant radiculopathy or neurological deficits. A conservative laminectomy offers direct access to the involved facet joint and associated epidural inflammatory changes for debridement and nerve root decompression. In our case, this type of exposure allowed smooth insertion of the epidural catheter with a Penfield No. 3 dissector. It is our opinion that PFJ decompression and concomitant SEA evacuation would also be achievable by inserting a fluoroscopically-guided epidural catheter through a minimally invasive tubular reactor system.

Multiple laminectomies have been reported for extensive SEA [17] but minimally invasive decompression, combined with catheter irrigation and drainage, may be preferable as it potentially reduces iatrogenic instability, postoperative pain, and blood loss [9, 11]. This is important in the management of SEA since it often afflicts patients with numerous comorbidities [18].

Several catheters for irrigation of the epidural space have been described, including the Fogarty catheter, pediatric feeding tube, and ventriculoperitoneal silicon shunt catheter [8, 11, 19, 20]. Concerns have been raised with the use of the Fogarty balloon because it may subject the neural elements to excessive hydraulic pressure [11]. Abd-El-Barr et al. [11] described the use of a pediatric nasogastric (NG) tube, which is smaller and more flexible than the Fogarty catheter. One drawback of the pediatric NG tube is its poor visualization on X-ray [11].

We used two different radio-opaque catheters to evacuate SEAs. We found that compared to the 5 French open-ended barium impregnated ureteral catheter (Cook®, Bloomington, IN), the 7 French TLS drain (Stryker®, Kalamazoo, MI) allows both irrigation and aspiration of the fluid component of the SEA. Although the ureteral catheter was too small to aspirate purulent discharge, its superior rigidity compared to the TLS drain makes it easier to be manually advanced under fluoroscopic guidance in the posterior epidural space. This may offer an advantage when more distant rigid pockets of fluid need to be reached and drained.

## Conclusions

A limited approach to the spine with the use of small radio-opaque catheters to effectively drain extensive SEAs can be achieved with less morbidity. The visualization of the catheter with fluoroscopy allows direct confirmation of the correct position of the catheter to achieve a complete evacuation of the SEA. We believe this technique offers superior safety and efficacy than an epidural catheter inserted blindly. This minimal approach still presents, however, the drawback of limited visualization of the entire abscess.
